# Strengthening bioinformatics education: e-learning initiatives across ELIXIR Nodes and Communities

**DOI:** 10.12688/f1000research.173116.1

**Published:** 2025-12-22

**Authors:** Monique Zahn-Zabala, Minna Ahokas, Bérénice Batut, Sebastian Beier, Alexander Botzki, Alexia Cardona, Kristina Gruden, Eija Korpelainen, Maria Lehtivaara, Brane Leskosek, Bogdan Mirăuță, Ajay Mishra, Cyril Pommier, Krzysztof Poterlowicz, Helena Rasche, Olivier Sand, Rob Waterhouse, Samantha Wittke, Cătălina Zenoaga-Barbăroșie, Mihail Anton, Daniel Wibberg

**Affiliations:** 1 Swiss Institute of Bioinformatics, Lausanne, Vaud, Switzerland; 2 CSC IT Center for Science Ltd, Espoo, Uusimaa, Finland; 3 Institut Francais de Bioinformatique, Villejuif, Île-de-France, France; 4 Universite Clermont Auvergne, Clermont-Ferrand, Auvergne-Rhône-Alpes, France; 5 Forschungszentrum Julich GmbH, Jülich, North Rhine-Westphalia, Germany; 6 Vlaams Instituut voor Biotechnologie, Ghent, Flanders, Belgium; 7 University of Cambridge, Cambridge, England, UK; 8 National Institute of Biology Department of Biotechnology and Systems Biology, Ljubljana, Ljubljana, Slovenia; 9 University of Ljubljana, Ljubljana, Ljubljana, Slovenia; 10 Romanian Bioinformatics Cluster, Târgu Mureș, Romania; 11 EMBL-EBI, Hinxton, England, UK; 12 Universite Paris-Saclay, Orsay, Île-de-France, France; 13 University of Bradford Faculty of Life Sciences, Bradford, England, UK; 14 Erasmus MC Department of Pathology and Clinical Bioinformatics, Rotterdam, South Holland, The Netherlands; 15 ELIXIR Hub, Hinxton, England, UK; 16 Chalmers University of Technology Department of Life Sciences, Gothenburg, Västra Götaland County, Sweden

**Keywords:** E-learning, Bioinformatics Training, Learning Management Systems, FAIR Principles

## Abstract

The rapid evolution of bioinformatics and data-driven life sciences necessitates widespread, effective training solutions capable of transcending geographical and institutional boundaries. ELIXIR, as a pan-European bioinformatics research infrastructure, has strategically embraced e-learning methodologies to meet this challenge. This white paper systematically reviews the current landscape of e-learning initiatives across various ELIXIR Nodes and Communities, detailing both historical developments and contemporary practices. It identifies core attributes and desirable features of effective e-learning, presenting an analysis of diverse educational platforms and the deployment of Learning Management Systems (LMS) within ELIXIR’s framework. Emphasis is placed on the interactive, open-access, and sustainable nature of these resources, exemplified by platforms such as the Training e-Support System (TeSS) and the ELIXIR-SI eLearning Platform (EeLP). The paper highlights critical advancements toward standardization and interoperability through initiatives such as the adoption of SCORM protocols, facilitating resource reuse across Nodes. Additionally, the integration of e-learning into broader educational strategies—such as hybrid learning environments and structured learning paths—is examined. Finally, future directions are discussed, including strategies for integrating e-learning with traditional training methods, enhancing trainer expertise, and further expanding the availability and FAIRification of bioinformatics training resources.

## Introduction

ELIXIR ^1^ is a pan-European research infrastructure within the European Strategy Forum on Research Infrastructures (ESFRI). Its mission is to build a sustainable infrastructure to support European life science research and its translation to medicine and the environment, the bio-industries and society. ELIXIR is an inter-governmental organisation which brings together existing bioinformatics resources. Coordinated by the ELIXIR Hub, its 23 members are European countries; the scientific community in each member country develops their national Node, which operates the services and resources that are part of ELIXIR. Each ELIXIR Node is itself a network of national life science organisations, coordinated by a lead institute. ELIXIR regroups experts in specific themes across ELIXIR Nodes and externally around Communities who drive the development of standards, services, and training in and across ELIXIR. It also concentrates its efforts around five central areas of activity, referred to as Platforms: Data, Tools, Compute, Interoperability and Training.

The ELIXIR Training Platform ^2^ (TrP) was established to develop a training community that spans all ELIXIR member states. The TrP aims to strengthen national training programmes, grow bioinformatics training capacity and competence across Europe, and empower researchers to use ELIXIR’s services and tools ( Alloza et al. 2024). To do so, the Platform establishes and implements best practices in bioinformatics training, through a T raining Toolkit. ^3^ Secondly, it supports training providers across Europe in creating and delivering training for developers, researchers and trainers. Thirdly, the TrP builds a sustainable training infrastructure. TeSS, ^4^ a one-stop shop platform of training events and materials for trainees, trainers, and training service providers, is part of this infrastructure ( Beard et al. 2020). The Nodes provide traditional face-to-face courses in the classroom, as well as online training events. They also provide materials for self-learning, ranging from videos, course materials, and e-learning.

## E-learning definition

The term “e-learning” has been subject to varied interpretations across different contexts, prompting ELIXIR and GOBLET trainers to engage in a collaborative workshop to establish a precise definition. The outcomes of this discussion were formalized in a report ( Attwood et al. 2016), which defined e-learning as “a mode of learning facilitated and supported by information and communications technologies, in which educational resources may be accessed via the Internet or intranets.” A distinguishing characteristic of e-learning is the absence of a trainer throughout the training and its availability online. This also applies to other self-training approaches, for instance educational materials online or videos. While the possibility for trainees to access educational material at any time and to go through it at their own pace is clearly an advantage, it requires that the trainee be motivated – something traditional textbooks also require.

Eight essential attributes of e-learning were defined by the trainers: (1) online, (2) open access, (3) interactive, (4) easy-to-use, (5) prerequisites, (6) measurable learning outcomes, (7) maintainable, and (8) sustainable. An additional seven attributes were judged to be desirable: (1) available on demand, (2) self-paced, (3) contextual, (4) quality control, (5) scalable, (6) interoperable, and (7) support. While many of these attributes also apply to traditional classroom teaching, it is the interactivity of the e-learning material which replaces the interactions with the trainer and other course participants. It is the inherent interactivity of e-learning which makes for an active learning process. In a recent survey, 63.2% of industry respondents and 84.1% of those in academia indicated that their preferred training format was “E-learning materials to consult anytime” ( Alves et al., 2023).

Several of the attributes make e-learning likely to reach a large audience: (1) open access, (2) contextual, (3) easy-to-use, (4) interactive, and (5) measurable learning outcomes. By eliminating the need for trainer availability and leveraging digital delivery, e-learning is particularly well-suited for capacity building. Once the technical aspects of creating e-learning courses have been mastered, trainers can efficiently make use of their time by adopting this approach, particularly when the course has already been taught many times. E-learning courses have the advantage that they can be followed by an infinite number of students anytime and anywhere. This gain in efficiency has led to widespread adoption of e-learning by institutions worldwide, including numerous ELIXIR Nodes.

## E-learning at the ELIXIR Nodes

The implementation of e-learning across ELIXIR Nodes has largely occurred through independent efforts, with several Nodes actively participating in the E-learning Interest Group. ^5^ This group convenes on a monthly basis to foster the exchange of best practices, address shared challenges, and advance the collective development of e-learning resources. To provide a better idea of the path taken by each Node in developing e-learning and its LMS of choice ( Table 1), each Node has provided a summary of its e-learning activities to date.

**Table 1.** ELIXIR Nodes offering e-learning courses. It summarises which Nodes provide e-learning (as defined above), the year the first e-learning was made available, the website(s) where the e-learning courses are listed, as well as the conditions to access the e-learning courses. There are currently 9 ELIXIR nodes which offer e-learning. EMBL-EBI was the first to do so in 2011, followed closely by the Swiss node and, over the years by the remaining nodes. The majority of Nodes have a single website from which trainees can choose to follow free e-learning courses (except for ELIXIR France). Login is not required to access the e-learning courses except for those of ELIXIR Belgium, Finland, Romania and Slovenia. Note that some Node institutes adopted e-learning prior to being part of the Node, or even prior to the creation of ELIXIR.

ELIXIR Node	First E-learning Course	Website(s) with E-learning Courses	Accessibility
ELIXIR-BE	2020	https://elearning.vib.be/	Login required; mostly free
ELIXIR-CH	2012	https://www.sib.swiss/training/e-learning	Open and free
ELIXIR-DE	2016	https://www.denbi.de/online-training-media-library	Open and free
ELIXIR-FI	2019	https://e-learn.csc.fi/	Login required; mostly free
ELIXIR-FR	2024	https://sandboxbio.france-bioinformatique.fr, https://ifb-elixirfr.github.io/Wasm4Learn/	Open and free
ELIXIR-RO	2024	https://elixir.mf.uni-lj.si/course/index.php?categoryid=33	Open and free, login required
ELIXIR-SI	2015	https://elixir.mf.uni-lj.si/	Login required; mostly free
ELIXIR-UK	2015	https://tess.elixir-europe.org/events?country=United+Kingdom https://tess.elixir-europe.org/materials?node=United+Kingdom&resource_type=e-learning	Multiple accessibility options
EMBL-EBI	2011	https://www.ebi.ac.uk/training/on-demand	Open and free

### ELIXIR Belgium (BE)

The VIB (Flemish Institute of Biotechnology) coordinates the activities of the ELIXIR Belgium Node’s endeavours. Embracing the digital age, VIB has pioneered an e-learning platform that complements the traditional classroom short-format courses. This platform primarily caters to novices, offering an array of beginner-level courses that serve as a foundation for future scientific research. The journey began with biosafety, where the first e-learning modules, crafted by external experts, laid the groundwork for what would become a robust educational framework. Since 2020, the VIB Training team has been diligently expanding this platform, introducing 2-3 new courses annually. The curriculum spans a diverse spectrum, delving into the basics of bioinformatics, guiding learners through the essentials of data management, and equipping them with vital programming and computing skills in Linux, Python using Dodona, ^6^ and High Performance Computing. Moreover, VIB upskills its internal talent by offering specialised introductory courses in Technology Transfer, Research Ethics and Data Analytics for Websites, ensuring a well-rounded educational experience that fosters both scientific and professional growth.

### ELIXIR Switzerland (CH)

The SIB Swiss Institute of Bioinformatics is the institute that oversees the work in the Node. Learner-centred education (e-learning, course material, videos) complement teacher-centred education – practically all e-learning courses offered are at the beginner level. The first e-learning courses developed at SIB were *ad hoc.* The first two courses were at the request of the Food and Agriculture Organization (FAO) of the United Nations, while the two additional courses were for new users of the Vital-IT Competence Centre in Bioinformatics and Computational Biology. Since 2021, the SIB has been creating 2-3 new courses every year. Most modules take 1 to 1.5 hours to complete, taking into account the normal attention span of trainees. The courses cover a wide range of topics, from topics in bioinformatics (BLAST, MSA, phylogenetics), data management (file formats and vocabularies) to introductions to SIB resources (ISMARA, Cellosaurus, SARS-CoV-2: Studying a new virus) and programming and computing techniques (UNIX, Python, High Performance Computing). The latest courses cover diverse aspects of data management and were developed as part of the CAS in Data Stewardship, University of Lausanne, 2024-2025. All SIB e-learning courses are found in TeSS.

### ELIXIR Germany (DE)

The German Network for Bioinformatics Infrastructure (de.NBI), which represents the German ELIXIR Node ( Tauch and Al-Dilaimi 2019), has been compiling a collection of training materials since 2017 ( Wibberg et al. 2020) and guidelines for the preparation of e-learning material were established in 2018. These resources are on a dedicated media library webpage which serves as a supplement to traditional training courses, offering a self-paced learning environment that is particularly well-suited for beginners. This library includes various e-learning modules, course materials, and instructional videos. The library serves as a vital supplement to traditional training courses, offering a self-paced learning environment that is particularly well-suited for beginners.

The content spans basic bioinformatics topics such as Research Data Management (RDM) ( Mayer et al. 2021) or R programming as well as a range of other de.NBI and ELIXIR-DE tools, e.g. Galaxy, Bioconductor. This library not only facilitates the training of researchers in the use of de.NBI tools. By providing easy and open access to training materials, the library essentially supports an unlimited number of participants, significantly scaling up the reach and impact of de. NBI training initiatives. The de.NBI’s commitment to education and training is evident in its continuous efforts to expand and update the media library, ensuring that it remains a valuable resource for the scientific community.

### ELIXIR Finland (FI)

The ELIXIR-Finland Node CSC organises a wide range of training in bioinformatics, AI and data analytics, High Performance Computing, programming and data management. Our online courses ^7^ are implemented in the Moodle-based eLena e-learning environment ^8^ and they can be accessed using the Life Science login. In addition, the programming and data analytics courses typically use our notebook environment Noppe, ^9^ which allows easy, installation-free studying.

Our bioinformatics e-learning courses include Single-cell RNA-seq data analysis ^10^ and Spatially-resolved transcriptomics (Visium) data analysis. ^11^ Each section of the courses contains lecture videos, hands-on exercises, and questions and tasks, which allow the participants to check if they have reached the learning goals. In order to enable non-coding scientists to analyse data, we use in the exercises the web based Chipster data analysis software (see also Chipster course materials, ^12^ including slides, exercises, example analysis sessions and lecture videos).

Our Research Data Management (RDM) Competence Center offers a wide range of webinars and tutorial videos on RDM, providing practical guidance to support researchers at every stage of the data lifecycle. The self-study Research Data Management ^13^ course, available on our e-learning platform, offers an introduction to key aspects of RDM, from planning and organising data to sharing and reusing it.

### ELIXIR France (FR)

The Institut Français de Bioinformatique (IFB) is a federation of regional platforms plus a coordination platform called IFB-core. The IFB Training team only started to work on e- l earning solutions ^14^ four years ago. The main motivation was to prepare life scientists for other training courses requiring Unix and R skills as prerequisites. For this, we wanted our e-learners to have access to environments emulating an actual system in their browser. Previously, students were directed towards DataCamp, ^15^ but a change in business model reduced the freely available e-learning courses. Katacoda ^16^ was then used but disappeared unexpectedly in July 2022. As the content was GitHub-based, it was quickly migrated to Killercoda ^17^ where it is available at https://killercoda.com/ifb-eformation-linux. Next, collaborating with the https://sandbox.bio owner led to the publication of our tutorials on Sandbox.bio at https://sandbox.bio/community/ and on our own instance at https://sandboxbio.france-bioinformatique.fr/. Impressed by the capabilities of the Web Assembly (Wasm) technology, we started to develop material on other topics (for instance, R programming) on a dedicated platform called Wasm4Learn and to work on the associated competency reference frames.

### ELIXIR Romania (RO)

Romania became an ELIXIR Observer in 2023 and is working toward full membership, with the Romanian Bioinformatics Cluster (CRB) as its official node. Since 2018, the Romanian Society of Bioinformatics (RSBI), a CRB founding member, has led national bioinformatics training efforts, organising a large number of bioinformatics workshops ( Mirăut¸ă et al. 2024). However, many of the training materials developed with these occasions were lost over time, limiting their reuse and hindering sustainable training.

To address this, CRB launched an initiative in 2024 to improve the FAIRification of bioinformatics training resources. The initial step was to directly adopt an existing solution developed by ELIXIR Nodes, the ELIXIR-SI eLearning Platform (see next section). An instance of this platform was developed for the Romanian community, ^18^ and was tested during two training events. However, an internal review revealed that the approach did not successfully engage the community.

Community option is crucial for such a platform to effectively achieve its objectives. Therefore, CRB launched a community consultation to assess: existing e-learning approaches with the Romanian research community, perspectives of training FAIRification, preferences for training material management systems and the level of interest in a national e-learning platform. Conducted in collaboration with the Open Science Knowledge Hub at the Executive Agency for Higher Education, Research, Development and Innovation Funding (UEFISCDI), the consultation gathered 222 responses, including 54 from life sciences.

While multiple institutional e-learning platforms exist in Romania, most are restricted to university-affiliated users, and 83% of respondents expressed a need for a national e-learning platform. Although this high positive rate may be influenced by survey biases, it indicates a strong need to provide community wide solutions. Additionally, the consultation assessed awareness and usage of ELIXIR-affiliated training resources. Galaxy Training was relatively well known and used (13 out of 54 respondents) whereas awareness of TeSS was extremely low (only 2 out of 54 respondents), suggesting that systematic engagement actions are needed to enhance awareness and visibility of training registries within the community.

### ELIXIR Slovenia (SI)

The ELIXIR-SI Node is based at University of Ljubljana, Faculty of Medicine. Within the Slovenian Node, the Centre ELIXIR-SI and its e-learning team is responsible for e-learning activities. This team initiated the establishment of the ELIXIR e-learning group within the project ELIXIR-EXCELERATE and which is now part of TrP. The Centre ELIXIR-SI team developed the ELIXIR-SI eLearning Platform ^19^ (EeLP), and one of the services offered by the ELIXIR TrP. Launched in 2015, EeLP is a Learning Management System (LMS) that provides storage for courses and training materials, including as an open e-learning tools and services platform for trainers and trainees. EeLP is a single access point with user management for different courses developed by members of ELIXIR network where all necessary information, dynamic educational resources, tools and services for any training event, including registration, embedded access to compute resources (HPC), exams, quizzes, feedback surveys, interactive H5P applications, communications and certificates among others are available. In EeLP learning paths can also be developed, e.g. as developed for the project PerMedCoE. ^20^ More about EeLP with examples of e-learning courses and features was recorded and is available as an ELIXIR webinar. ^21^

### ELIXIR United Kingdom (UK)

ELIXIR-UK is a distributed network of 28 (and growing) research-performing organisations, representing national expertise in data-driven life sciences and providing a platform for UK bioinformatics resources to engage with ELIXIR. As part of its e-learning initiatives, ELIXIR-UK members have developed a wide range of courses and training materials tailored for the higher education and research community, with a strong focus on Research Data Management (RDM), data science, and bioinformatics.

A key achievement in this space is the ELIXIR-UK flagship Data Stewardship Training Fellowship, which has facilitated the creation of diverse e-learning resources, including materials within the Galaxy Training Network and concise video-based courses such as RDMbites. Additionally, the Training e-Support System (TeSS) ^22^ - an ELIXIR-UK Node service - plays a crucial role in aggregating and increasing the visibility of e-learning courses across different ELIXIR Nodes.

Beyond content development, ELIXIR-UK actively contributes to community building and capacity development. It co-leads the ELIXIR Learning Paths Focus Group, ^23^ which works to enhance educational strategies by developing structured learning programmes and supporting their implementation. ELIXIR-UK also co-leads the ELIXIR-GOBLET Train-the-Trainer programme, ^24^ which fosters a collaborative network of trainers, providing opportunities for reciprocal support and shared best practices through the TtT instructors’ community.

### EMBL-EBI

EMBL-EBI provides a range of training across the field of data-driven life sciences to guide researchers in both accessing open data using EMBL-EBI data services and analysing their own data. The EMBL-EBI Training website ^25^ follows FAIR principles to improve user experience, ensuring content is findable, accessible, interoperable, and reusable ( Swan et al. 2024). Features include keyword search, themed collections, and open access without registration, with optional login for allowing users to create their personalised learning profile. The site supports various devices and links competencies to courses, promoting resource reuse and knowledge sharing. E-learning is provided through the on-demand training section ^26^ where two major components are online tutorials and collection.

Tutorials are designed for self-paced learning and cover a broad spectrum of topics in data science. They range from beginner-level tutorials like Bioinformatics for the terrified, ^27^ to more advanced tutorials focusing on specific biological domains or EMBL-EBI data services such as AlphaFold: A practical guide ^28^ or GWAS Catalog: Exploring SNP-trait associations. ^29^ These tutorials incorporate recorded videos and interactive elements created with the H5P application ^30^ to engage learners, enhance their learning experience, and support self-assessment.

Curated collections of on-demand training materials, including recorded webinars and course materials, bring together a variety of training on a specific topic, such as Biostatistics ^31^ and Data-driven plant sciences ^32^ to guide learners through relevant on-demand training.

## E-learning in ELIXIR communities

This section provides an overview of the diverse array of educational resources available across various ELIXIR Communities – groups of experts across ELIXIR Nodes and externally that represent a scientific or technological theme, and drive the development of standards, services, and training in and across ELIXIR.

### ELIXIR biodiversity community

Fostering knowledge transfer in biodiversity data management and analysis is one of the five main goals guiding the work of the ELIXIR Biodiversity Community. The aims are to: (1) support community-driven skills sharing focused on understanding how to benefit from the use of available standards and best practices; (2) connect developers of tools/workflows/databases with user communities through training that responds to changing technologies and associated services; and (3) expose collections of biodiversity-related training materials, for example through TeSS, the Galaxy Training Network, and RDMkit.

Actions to develop and share training resources that embrace at least some of the essential or desirable attributes that define e-learning are to date largely driven by initiatives with which the ELIXIR Biodiversity Community interacts. A key example from the biodiversity genomics domain is the Knowledge Hub ^33^ developed and maintained by the European Reference Genome Atlas (ERGA) initiative ( Mazzoni et al. 2023). The ERGA Knowledge Hub is a platform for collecting, organising, and sharing openly accessible educational resources related to biomaterial sampling, reference genome sequencing, assembly, annotation, and analysis, as well as for data management, communications and skills sharing, and legal and ethical considerations.

A goal of the ELIXIR Biodiversity Community is to continuously improve offerings of the ERGA Knowledge Hub and other platforms by supporting and encouraging the development of more training content that delivers rich e-learning experiences.

### ELIXIR galaxy community

The ELIXIR Galaxy Community has integrated e-learning as a key aspect of its training strategy, aligning with ELIXIR’s mission to build bioinformatics skills and data management capacity across Europe. The Galaxy Project offers an accessible platform for large-scale life sciences data analysis, complemented by interactive, on-demand training materials ( The Galaxy Community 2024). These resources empower users—ranging from beginners to advanced learners—to enhance their skills independently and at their own pace, without the need for face-to-face instruction.

The Galaxy Training Network (GTN) ( Hiltemann et al. 2023) provides over 400 tutorials on diverse topics, including bioinformatics tools, visualization, didactics, and FAIR data management. Training materials cater to learners at various levels: beginner (46%), intermediate (45%), and advanced (8%). Freely available to the global bioinformatics community, these resources promote accessibility and scalability.

Galaxy’s e-learning adheres to ELIXIR’s essential attributes of interactivity, open access, and sustainability. Materials include SMART learning objectives, self-assessment tools, and automated quality checks. Metadata compliance ensures interoperability and discoverability through platforms like ELIXIR TeSS. By offering flexible, high-quality resources, the Galaxy Community supports a global audience in advancing their bioinformatics expertise.

### ELIXIR plant sciences community

The Plant Sciences Community is an interdisciplinary group of researchers who aim to develop and leverage tools, databases, standards and best practices for plant research ( Pommier et al. 2021). The Community addresses diverse learning needs through various training formats, including virtual workshops, and self-guided resources like videos and written guides.

To ensure broad and effective adoption of the Plant Community Service Bundles, interactive e-learning materials have been established, accessible through both TeSS ( https://tess.elixir-europe.org/materials/workshop-on-resources-for-plant-sciences-202) and the ELIXIR Slovenia E-Learning Platform (EeLP) ( https://elixir.mf.uni-lj.si/course/view.php?id=107). This material is specifically designed to support users at every stage of their learning journey, catering to diverse learning preferences with a blend of multimedia and interactive hands-on content.

To anchor learning in real-world applications, an engaging and interactive CorkOak tutorial ( https://corkoak-usecase.readthedocs.io/en/latest/) places users in a practical plant science scenario. Here, participants actively apply Service Bundle tools to address different exercises representing research problems, guided through every step by both video and written instructions. This case study bridges theory and practice, giving users the confidence to implement new tools in their own work.

By weaving together interactive modules, detailed documentation, and practical exercises, the Plant Community Service Bundles e-learning suite offers an engaging, adaptable, and effective learning environment—equipping plant scientists across ELIXIR and beyond to confidently use new tools and approaches in their research.

### ELIXIR Single-Cell Omics community

The goals of the ELIXIR Single-Cell Omics (SCO) Community are to identify the critical challenges in the rapidly evolving field of single-cell and spatial omics data, as well as to bring together international efforts so as to address the needs of the research community. As described in their white paper ( Czarnewski et al. 2022), training efforts will focus on upskilling scientists in SCO data analysis and standards. The Community will coordinate with the TrP, and particular efforts will be made to make the training scalable and FAIR. The SCO Community recently conducted a recent training survey and compiled a list of course instances. ^34^ The training materials, including slides and exercises for these courses are available online, with the recorded lectures for some of these courses also available. The videos and coding examples from the perturbation modeling AHM workshop will be part of an e-learning module.

## Summary

The development and inclusion of e-learning reflects the dynamic progression of educational technology, with numerous life science data hubs enhancing the quality, accessibility, and relevance of these learning resources. E-learning is defined as education supported by digital technologies, accessible online or via intranets, characterized by its self-paced, open-access and interactive nature. While e-learning and open-access, online training materials are both suitable to increase training capacity compared to traditional trainer-lead courses, e-learning distinguishes itself by its interactive approach, for instance through the inclusion of quizzes which test the learners’ knowledge and provide feedback on wrong answers.

Organizations and institutions, including ELIXIR Nodes and Communities, have independently developed a wide range of e-learning resources, from beginner-level courses to advanced modules covering bioinformatics, data management, and programming. EMBL-EBI and ELIXIR Switzerland pioneered an e-learning offering more than 13 years ago. Now, EMBL-EBI provides FAIR training in data-driven life sciences, including self-paced tutorials and curated collections. ELIXIR Switzerland’s offering has recently expanded by 2-3 new courses every year, providing a comprehensive offering in bioinformatics, programming and computing topics, data management and SIB resources. In 2015, ELIXIR Slovenia set up the ELIXIR-SI eLearning Platform (EeLP) platform developing comprehensive LMS services over the years. Only a year later, ELIXIR Germany embarked with a growing media library of e-learning materials. Starting in 2019, ELIXIR Finland offers e-learning courses which now cover topics in bioinformatics, AI, data analytics, High Performance Computing, and programming on Moodle and Noppe. From 2020 onwards, ELIXIR Belgium has expanded its e-learning offering starting with beginner-level bioinformatics courses to include complementary topics like Technology Transfer and Research Ethics. In the last four years, ELIXIR France explored several hosting solutions for e-learning material like Killercoda and Sandbox.bio as well as developing new materials on Wasm4Learn. In 2024, ELIXIR Romania launched an initiative to improve the FAIRification of training resources. The e-learning offering of ELIXIR UK focuses on Research Data Management, data science, and bioinformatics, co-leading educational strategy groups. All of these e-learning materials of ELIXIR nodes are findable via the e-learning tab in TeSS.

The ELIXIR communities are actively engaged in enhancing bioinformatics skills through diverse online learning resources. The Biodiversity Community supports community-driven skills sharing and connects developers with user communities through targeted training, maintaining the ERGA Knowledge Hub for sharing educational resources on biodiversity genomics. The Galaxy Community offers over 400 tutorials on bioinformatics tools, visualization, and FAIR data management through the Galaxy Training Network, promoting accessibility and scalability. The Plant Sciences Community conducts the FONDUE Datathon to train participants in submitting plant genomic data to EBI repositories and introduces Service Bundles for comparative genomics, multi-omics data analysis, and phenotyping data publication. The Single-Cell Omics Community focuses on upskilling scientists in single-cell and spatial omics data analysis and standards, providing a range of training materials, including slides, exercises, and recorded lectures, available online to support the research community in addressing critical challenges in this rapidly evolving field.

## Future directions

Efforts to make e-learning resources FAIR—Findable, Accessible, Interoperable, and Reusable—align with the broader mission of making life-science data more accessible and collaborative. The adoption of best practices, such as adhering to standardization through ontologies and developing certification frameworks, ensures that materials harmonize with the efforts of the global scientific community. Looking ahead, strategic documents like white papers could consolidate current achievements in e-learning while addressing challenges related to standardization and technological integration. These initiatives collectively strengthen the e-learning strategy, positioning it as a leader in delivering premier, forward-thinking educational resources to the life-science community.

While e-learning is being developed and offered in a number of ELIXIR Nodes, there are many Nodes which have yet to do so. These nodes may wish to use the ELIXIR-SI eLearning Platform (EeLP) to create and host their e-learning courses, rather than invest in developing their own solution. To give these Nodes a jump start, an e-learning course ‘ ELIXIR e-learning definitions and guidelines for e-learning course development ^35^’ is available. Obviously, they are welcome in the ELIXIR E-learning Interest Group where they can discuss with members of the other Nodes and get up to speed. Once they have created their e-learning courses on the platform, the ELIXIR e-learning curation guideline takes them step-by-step through the process of annotating their courses for inclusion in TeSS ( Zahn et al. 2023).

The ELIXIR Nodes and Communities have developed their e-learning content independently, using different Learning Management Systems (LMS). However, reusable e-learning materials require these to be interoperable, i.e. in a standard format supported by the Learning Management Systems (LMSs) being used. Sharable Content Object Reference Model ( SCORM) ^36^ is a set of standards for e-learning which is recognized internationally. It is a technical standard which describes how online learning content and LMSs communicate with each other. SCORM is compatible with the free and open-source Moodle, ^37^ ILIAS, ^38^ and Sakai ^39^ LMSs. The plugin Tin Canny Reporting can be used to work with SCORM packages on Wordpress, ^40^ the LMS used by the EMBL-EBI Node. Initial tests to see whether content created in one Node and exported in the SCORM format could be integrated and modified in LMSs elsewhere were carried out. ELIXIR Belgium created a SCORM package based on the markdown content with the LiaScript exporter. ^41^ The ZIP file was successfully imported in the ELIXIR Switzerland Moodle instance. One can thus envision a not-too distant future in which e-learning courses from different Nodes can be combined to meet training requirements, effectively collaborating in an efficient manner.

A new development in the training field is to combine e-learning and trainer-led courses. E-learning has significantly enhanced ELIXIR’s training offerings as these have been integrated in institutional onboarding trajectories in ELIXIR UK, and soon in Belgium. The implementation of e-learning as a pre-learning trajectory for, e.g. the ‘Statistics using R’ course in ELIXIR-BE, led to new course offerings integrating several modalities after the pandemic and encouraged trainers to professionalize their skills in creating e-learning compatible activities. These activities can be reused in in-person training, ensuring consistency and quality. Additionally, the future development of learning paths with e-learning components in ELIXIR Switzerland promises to provide structured and comprehensive training experiences. These approaches not only maximize resource efficiency but also offer flexible, accessible learning opportunities for participants across different regions.

Looking ahead, several initiatives are poised to shape the future of e-learning. A key initiative involves the development of Train-the-Trainer programs aimed at equipping educators with the skills necessary for effective online teaching. These programs emphasize the importance of interactive, engaging content that is pedagogically sound and technically robust, catering to diverse learner needs, criteria which also apply to e-learning.

## Data Availability

No data is associated with this article.

## Footnotes

^1^

https://elixir-europe.org

^2^

https://elixir-europe.org/platforms/training

^3^

https://elixir-europe.org/internal-projects/commissioned-services/training-toolkit

^4^

https://tess.elixir-europe.org

^5^

https://elixir-europe-training.github.io/ELIXIR-Training-SPLASH/elearning

^6^

https://dodona.ugent.be

^7^

https://csc.fi/en/trainings/online-training

^8^

https://e-learn.csc.fi

^9^

https://noppe.2.rahtiapp.fi/welcome

^10^

https://e-learn.csc.fi/course/view.php?id=17

^11^

https://e-learn.csc.fi/course/view.php?id=146

^12^

https://chipster.2.rahtiapp.fi/manual/courses.html

^13^

https://csc.fi/en/training-calendar/cscs-self-study-research-data-management-course

^14^

https://www.france-bioinformatique.fr/en/e-learning

^15^

https://www.datacamp.com

^16^

https://www.katacoda.com

^17^

https://killercoda.com/learn

^18^

https://elixir.mf.uni-lj.si/crb

^19^

https://elixir.mf.uni-lj.si

^20^

https://elixir.mf.uni-lj.si/permedcoe

^21^

https://elixir-europe.org/events/elixir-webinar-elixir-sl-elearning-platform

^22^

https://tess.elixir-europe.org

^23^

https://elixir-europe.org/focus-groups/learning-paths

^24^

https://elixir-europe.org/platforms/training/train-the-trainer

^25^

https://www.ebi.ac.uk/training

^26^

https://www.ebi.ac.uk/training/on-demandhttps://www.ebi.ac.uk/training/on-demand

^27^

https://www.ebi.ac.uk/training/online/courses/bioinformatics-terrified

^28^

https://www.ebi.ac.uk/training/online/courses/alphafold

^29^

https://www.ebi.ac.uk/training/online/courses/gwas-catalogue-exploring-snp-trait-associations

^30^

https://h5p.org

^31^

https://www.ebi.ac.uk/training/online/courses/biostatistics-introduction

^32^

https://www.ebi.ac.uk/training/online/courses/data-driven-plant-sciences

^33^

https://knowledge.erga-biodiversity.eu

^34^

https://www.singlecellomics.org/pages/training/index#training-materials

^35^

https://elixir.mf.uni-lj.si/course/view.php?id=10

^36^

https://en.wikipedia.org/wiki/Sharable_Content_Object_Reference_Model

^37^

https://moodle.org

^38^

https://www.ilias.de

^39^

https://www.sakailms.org

^40^

https://wordpress.org

^41^

https://liascript.github.io
