# Supplementary material for: Strengthening bioinformatics education: e-learning initiatives across ELIXIR Nodes and Communities
Source: F1000Res. 2025 Dec 22;14:ELIXIR-1422. [Version 1] doi: 10.12688/f1000research.173116.1 (PMC12831047; doi:10.12688/f1000research.173116.1)
Supplement: Peer review history

# Reviewer report: Reviewer response for version 1

**DOI:** 10.5256/f1000research.190899.r444757

**Published:** 2026-02-24

**Authors:** Anna-Sophie Fiston-Lavier

**Affiliations:** 1 Univ Montpellier, Paris, France

This article provides a comprehensive overview of the e-learning initiatives developed across ELIXIR Nodes and Communities.

 The topic is highly relevant, given the rapid evolution of bioinformatics and the growing need for FAIR training solutions. The manuscript brings together a list of practical resources and initiatives from across Europe that offers a valuable snapshot of the current e-learning landscape within ELIXIR.

 One of the main strengths of the article is its broad and inclusive scope. The authors successfully document e-learning activities across multiple ELIXIR Nodes and examples of thematic Communities. Such a way, they offer concrete examples of platforms, pedagogical approaches, and technical solutions. The structured presentation of Node-specific initiatives in Europe are listed in the Table 1. While each Node is presented, a broader summary table is missing. It is not possible to compare the Nodes as they do not all provide the same type of resources for the same group of people. Summarising the diversity of the Nodes will help readers to find the information they need.

 The manuscript also provides a clear and well-referenced definition of e-learning in the ELIXIR context, grounded in prior community discussions (Attwood et al., 2016). The distinction between essential and desirable attributes of e-learning resources is particularly useful and offers a practical framework that can guide future course development. The emphasis on FAIR principles, interoperability (e.g. SCORM adoption), and reuse of training materials is well aligned with ELIXIR’s broader strategic goals.

 Finally, the discussion of future directions, including hybrid learning models, learning paths, and Train-the-Trainer initiatives, adds value by situating current efforts within a longer-term vision for sustainable training infrastructure.

 While the article is rich in descriptive detail, it would benefit from a more explicit synthesis across Nodes and Communities. For example, a short comparative analysis highlighting common challenges (e.g. sustainability, learner engagement, maintenance costs, assessment of learning outcomes) and successful mitigation strategies would strengthen the manuscript and help readers extract transferable lessons.

 In addition, although many platforms and courses are described, there is limited discussion of evaluation metrics or evidence of impact. Where available, even qualitative indicators such as learner numbers, completion rates, or user feedback could help substantiate claims about effectiveness and scalability.

 The manuscript could also clarify more explicitly how the proposed essential and desirable attributes are currently met across the described initiatives. A concise mapping or summary indicating which attributes are systematically addressed and which remain aspirational would improve coherence between the conceptual framework and the descriptive sections.

 Finally, given the rapid pace of technological change, a brief discussion of potential risks (e.g. platform dependency, long-term maintenance of interactive content, or trainer workload) would provide a more balanced perspective.

 Overall, this article represents a valuable and well-documented contribution to the bioinformatics training literature and will be of interest to training coordinators, infrastructure developers, and policy makers within and beyond ELIXIR. With minor revisions to strengthen synthesis and discussion of impact, the manuscript is suitable for indexing and provides a solid reference point for future developments in e-learning within large research infrastructures.

 Maybe it has been already discuss but a table summary will be appreciated to highlight the different services and type of resources provided by each Node.

 How to assess the « quality »? enhancing the quality, accessibility, and relevance of these learning resources.

Is the topic of the opinion article discussed accurately in the context of the current literature?

Yes

Are arguments sufficiently supported by evidence from the published literature?

Yes

Are all factual statements correct and adequately supported by citations?

Yes

Are the conclusions drawn balanced and justified on the basis of the presented arguments?

Yes

Reviewer Expertise:

Bioinformatics, teaching innovation

I confirm that I have read this submission and believe that I have an appropriate level of expertise to confirm that it is of an acceptable scientific standard.

# Reviewer report: Reviewer response for version 1

**DOI:** 10.5256/f1000research.190899.r444751

**Published:** 2026-01-23

**Authors:** Gangqing Hu

**Affiliations:** 1 West Virginia University, Morgantown, WV, USA

The manuscript by Zahn-Zabala summarized the development of ELIXIR Nodes and Communities for bioinformatics e-learning. It discussed platforms such as TeSS and Eelp for interactivity, accessibility, and sustainability of the developed resources. The authors also provided overview of integration of these eLearning resources to broader educational schemes. I have the following points for the authors to consider.

 1. While it is understandable ELIXIR from Europe is the focus of the manuscript, the introduction will be benefit by highlighting similar bioinformatics e-learning portals in other places such as the NCBI Learn Portal, DNA Subway from CyVerse, Genomics workshop from Carpentry to name a few. How e-learning portals from ELIXIR nodes are complementary to these resources?

 2. The manuscript has described offering by each Node in detail. However, it remains unclear how effectiveness is evaluated. Table 1 could add additional columns to include main features of each node and ways of evaluation.

 3. ELIXIR Nodes and Communities are complementary. A diagram showing the relationship between ELIXIR Nodes and Communities will help reader to better appreciate the e-learning landscape of ELIXIR.

 4. The recent AI development, in particular LLM, has the potential to transform bioinformatics education. One of the ELIXIR (FI) does have an AI component. How ELIXIR account for this LLM possibility, which could be a worthy point to discuss as future directions.

Is the topic of the opinion article discussed accurately in the context of the current literature?

Yes

Are arguments sufficiently supported by evidence from the published literature?

Yes

Are all factual statements correct and adequately supported by citations?

Yes

Are the conclusions drawn balanced and justified on the basis of the presented arguments?

Yes

Reviewer Expertise:

bioinformatics, sequencing data analysis, LLMs in bioinformatics and medical informatics

I confirm that I have read this submission and believe that I have an appropriate level of expertise to confirm that it is of an acceptable scientific standard, however I have significant reservations, as outlined above.

# Reviewer report: Reviewer response for version 1

**DOI:** 10.5256/f1000research.190899.r444750

**Published:** 2026-01-21

**Authors:** Ming Chen

**Affiliations:** 1 Zhejiang University, Hangzhou, China

This opinion article systematically reviews e-learning initiatives for bioinformatics education across 9 ELIXIR Nodes and 4 Communities. It defines the core and desirable attributes of effective e-learning (e.g., open access, interactivity, interoperability), highlights key platforms (e.g., TeSS, EeLP) and best practices (e.g., adoption of SCORM standards for resource reuse), maps regional implementation efforts, identifies gaps in cross-node coordination and global outreach, and outlines future directions for standardization and capacity building.

 The work fills a critical need for centralized documentation of European bioinformatics e-learning resources. therefore, I would like to recommend its indexing in F1000 Research. However, bioinformatics e-learning is a cross-country trend, the article fails to engage with global literature on the topic and lacks non-European perspectives and comparative global evidence—particularly insights from emerging economies (e.g., China, India) that offer complementary models. a notable example is China's  education reform "101 Plan", which features robust cross-regional collaboration mechanisms and AI-enhanced teaching tools; personally I am the lead expert in bioinformatics education for this program).

 Additionally, the article requires supplementary citations for statistical claims and unvalidated factual statements.

Is the topic of the opinion article discussed accurately in the context of the current literature?

Yes

Are arguments sufficiently supported by evidence from the published literature?

Yes

Are all factual statements correct and adequately supported by citations?

Partly

Are the conclusions drawn balanced and justified on the basis of the presented arguments?

Yes

Reviewer Expertise:

Bioinformatics

I confirm that I have read this submission and believe that I have an appropriate level of expertise to confirm that it is of an acceptable scientific standard.

# Reviewer report: Reviewer response for version 1

**DOI:** 10.5256/f1000research.190899.r444758

**Published:** 2026-01-16

**Authors:** Michelle D. Brazas

**Affiliations:** 1 Informatics and Bio-computing, Ontario Institute for Cancer Research, Toronto, ON, Canada

This paper provides an overview and history of e-learning activities across ELIXIR nodes. The paper is informative, but could be much more informative if it provided guidance on lessons learned over the course of establishing e-learning resources. For example, how do ELIXIR nodes avoid redundant e-learning content? Its seems all nodes developed RDM materials - why? It could also be much more informative if it expanded on future directions.

 Other comments that need corrections include:

 - Table 1 website column - Not every link is to an LMS, so text below table should not refer to "LMS of choice"

 - Many abbreviations are repeated throughout document. The entire paper needs to be read and updated by a single author as it currently reads like all authors wrote a section and the sections do not connect to each other. Each node talks about different aspects of e-learning for example.

 - Aren't all ELIXIR e-learning materials in TeSS? Correct statement " All SIB e-learning courses are found in TeSS" or remove.

 - German paragraph has repeated text that should be removed

 - German paragraph has a hanging sentence "This library not only facilitates..."

 - Finland paragraph - please define CSC abbreviation

 Some aspects of the paper are so ELIXIR focused that external non-European reviewers cannot follow. This reviewer does not understand "The videos and coding examples from the perturbation modeling AHM workshop will be part of an e-learning module".

Is the topic of the opinion article discussed accurately in the context of the current literature?

Yes

Are arguments sufficiently supported by evidence from the published literature?

Yes

Are all factual statements correct and adequately supported by citations?

Yes

Are the conclusions drawn balanced and justified on the basis of the presented arguments?

Partly

Reviewer Expertise:

bioinformatics training

I confirm that I have read this submission and believe that I have an appropriate level of expertise to confirm that it is of an acceptable scientific standard, however I have significant reservations, as outlined above.
